# Dissecting fine-flavor cocoa bean fermentation through metabolomics analysis to break down the current metabolic paradigm

**DOI:** 10.1038/s41598-021-01427-8

**Published:** 2021-11-09

**Authors:** Fabio Herrera-Rocha, Mónica P. Cala, Jenny Lorena Aguirre Mejía, Claudia M. Rodríguez-López, María José Chica, Héctor Hugo Olarte, Miguel Fernández-Niño, Andrés Fernando Gonzalez Barrios

**Affiliations:** 1 Grupo de Diseño de Productos Y Procesos (GDPP), Departamento de Ingeniería Química Y de Alimentos, Universidad de los Andes, 111711 Bogotá, Colombia; 2 MetCore - Metabolomics Core Facility. Vice-Presidency for Research, Universidad de los Andes, Bogotá, Colombia; 3 CasaLuker S.A., Bogotá, Colombia; 4 Department of Bioorganic Chemistry, Leibniz-Institute of Plant Biochemistry, Weinberg 3, 06120 Halle, Germany

**Keywords:** Metabolomics, Plant sciences

## Abstract

Cocoa fermentation plays a crucial role in producing flavor and bioactive compounds of high demand for food and nutraceutical industries. Such fermentations are frequently described as a succession of three main groups of microorganisms (i.e., yeast, lactic acid, and acetic acid bacteria), each producing a relevant metabolite (i.e., ethanol, lactic acid, and acetic acid). Nevertheless, this view of fermentation overlooks two critical observations: the role of minor groups of microorganisms to produce valuable compounds and the influence of environmental factors (other than oxygen availability) on their biosynthesis. Dissecting the metabolome during spontaneous cocoa fermentation is a current challenge for the rational design of controlled fermentations. This study evaluates variations in the metabolic fingerprint during spontaneous fermentation of fine flavor cocoa through a multiplatform metabolomics approach. Our data suggested the presence of two phases of differential metabolic activity that correlate with the observed variations on temperature over fermentations: an exothermic and an isothermic phase. We observed a continuous increase in temperature from day 0 to day 4 of fermentation and a significant variation in flavonoids and peptides between phases. While the second phase, from day four on, was characterized for lower metabolic activity, concomitant with small upward and downward fluctuations in temperature. Our work is the first to reveal two phases of metabolic activity concomitant with two temperature phases during spontaneous cocoa fermentation. Here, we proposed a new paradigm of cocoa fermentation that considers the changes in the global metabolic activity over fermentation, thus changing the current paradigm based only on three main groups of microorganism and their primary metabolic products.

## Introduction

Cocoa fermentation is an essential step in chocolate production^1,2^. This process is critical for the biosynthesis of relevant flavors and bioactive compounds that determine the chocolate quality^2–4^. Incomplete/shorter fermentations result in cocoa with undesirable astringent flavors^3^, while overfermentations result in undesirable hammy and putrid flavors^5,6^. Despite their high relevance, very little information is available regarding the biochemistry and microbiology of cocoa fermentation. Additional research is still required to elucidate the synthesis of valuable compounds during this critical step in chocolate production.

Cocoa fermentation is a highly complex spontaneous process led by the interaction of heterogeneous microbial populations. Even though it is generally accepted that the origin of this microbial community is the environment(e.g., insects, worker hands, tools, and air) the exact origin of every microorganism group present in the coco fermentation has not been proven so far^3,7^. For this reason, the origin of the microbial community accountable for cocoa fermentation is still unclear^3,8,9^. Three significant phases during spontaneous cocoa fermentations have been previously proposed^10–13^ due to the interactions between three main groups of microorganisms (yeast, lactic acid bacteria, and acetic acid bacteria) and their metabolic products^9,14^. An initial anaerobic phase occurs at the beginning of the fermentation, in which yeasts produce ethanol using the fermentable sugars from the cocoa pulp^9^. Then, when the pulp begins to disappear and oxygen penetrates at a higher rate the fermentation mass, a second (aerobic) phase occurs. In this phase, Lactic Acid Bacteria (LAB) utilizes the remaining sugars (mainly fructose) to produce lactic acid that accumulates^9,15^. Finally, the last phase occurs when acetic acid bacteria (AAB) convert ethanol into acetic acid that strongly affects the microbial diversity of fermentation^9,15,16^. Based on this paradigm, different mathematical models of cocoa fermentation have been previously proposed to further study the dynamics among these phases^10–13^. Apart from the general metabolic activity, other specific biochemical processes in cocoa fermentation have been modeled. For instance, genome-scale reconstructions for selected strains of acetic acid and lactic acid bacteria isolated from cocoa fermentations have been proposed in various studies, even using advanced techniques as fluxomic to improve the models^17–19^.

However, recent metagenomics studies highlighted the relevance of other dominant groups of microorganisms during cocoa fermentations^8,13,14,20–23^. The results of these studies question the traditional view of cocoa fermentation. For instance, the dominance of other microorganisms such as *Bacillus*, *Pseudomonas, Aspergillus, Malassezia, and Pestalotiopsis* at several phases during cocoa fermentations have been recently demostrated^8,13,14,20–23^*.* The role of these microorganisms and their potential to produce valuable flavor and bioactive compounds during spontaneous cocoa fermentation still have to be elucidated. Therefore, a current essential challenge in cocoa research is identifying metabolites associated with these microorganisms to better understand their relevance during cocoa fermentation.However , only a few studies have dissected the variation in the metabolic profile during spontaneous cocoa fermentations^24–28^. Most of these studies focus on identifying metabolites during the fermentation of bulk cocoa (i.e., cocoa varieties with an ordinary flavor profile). In contrast, these kinds of analyses are rare and still in their infancy for fine-flavor cocoa. In this regard, to understand the metabolic dynamic of fine-flavor cocoa fermentation is a necessary previous step to connect the metabolites and microorganisms involved in this process.

Compared to bulk cocoa, fine-flavor offers a higher diversity of flavor attributes of high demand by the elite chocolatiers. Therefore, identifying changes in the metabolic fingerprint during spontaneous fermentation of fine-flavor cocoa is highly relevant to standardize this process and produce a high-quality bar of chocolate. Consequently, the goal of this study was to analyze the changes in the metabolic fingerprint during spontaneous fermentation of fine-flavor cocoa. Our data revealed two main phases of differential metabolic activity during spontaneous fermentation of fine-flavor cocoa that correlated with the observed variations on temperature and highlighted the relevance of comprehensive metabolomics studies to break down the current cocoa fermentation paradigm.

## Experimental section

### Cocoa beans fermentation

Cocoa fermentations were performed at the Luker Farm (Caldas, Colombia (5°4' N 75°41' W)) owned by CasaLuker S.A. Cocoa beans, from a standard, pre-designed, and a frequently-used mixture of Theobroma cacao clones (i.e., LUKER40, FSV41, FSA13, and TSH565), were selected for wooden box fermentations. Additional information regarding the used clones is available at the International Cocoa Germplasm Database (ICGD) (http://www.icgd.reading.ac.uk/).

Two independent fermentation boxes containing 400 kg of cocoa pulp-bean mass each was arranged using a ladder system in a pre-designed fermentation room as previously described^13^. Briefly, this fermentation room has an area of 84 m^2^ with a metallic ceiling and acrylic walls to prevent air current entering the fermentation zone. The average temperature and during day time are 33 °C while at night time is 25 °C. Humidity is around 60% with a maximum level of 94%. The fermentation mass was mixed from the fourth day on, every 48 h,to allow aeration . Standard cutting tests were used to evaluate the fermentation quality over time and determine its final point following the standard protocols^13^.

### Fermentation mass sampling

Three biological replicates, each consisting of 10 seeds, were collected from different fermentation mass locations—two of them from one fermentation box and the third one from the other fermentation box, as previously described^13^. Sampling was made at the beginning and every 24 h until the end of fermentation. To avoid chemical degradation, samples were frozen immediately at − 80 °C after collection. Furthermore, to connect the medium conditions and metabolic activity, we recorded the fermentation cocoa mass temperature variation every 4 min for each fermentation box using precision sensors placed in the center of the fermentation boxes.

This sampling process followed the guidelines and legislation settled by the ministry of environment and sustainable development of Colombia. We obtained a permission to access genetic and derivate resources according to resolution No 284 of 2020.

### Sample preparation

Fermentation samples were initially milled using a clean and precooled coffee grinder. Aliquots of 100 mg were further macerated in the presence of liquid nitrogen and subsequently defatted by the addition of 500 µL of n-hexane and vigorous vortex as previously reported^29^. Defatting was repeated three times for each sample to maximize the lipid removal. Metabolite extraction was performed by adding 1 mL of methanol-ultrapure water (70:30) to each sample. Then these extracts were placed into an ultrasonic bath at room temperature for 10 min following by vortex-mixed for 10 min. After that, the samples were centrifuged at 6000 g, 25 °C for 10 min, and the supernatant was collected for LC-QTOF-MS and GC-QTOF-MS analysis. For LC-QTOF-MS analysis, 30 µL of supernatant was mixed with 70 µL of Milli Q water and transferred to LC–MS vials. For GC-QTOF-MS, 30 µL of supernatant was evaporated to dryness using a SpeedVac concentrator system. Methoxymation was performed by adding 20 µL of *O*-methoxyamine hydrochloride (30 mg/mL in pyridine) to each sample and vigorously vortex-mixed 5 min. Then, samples were incubated at 70 °C for 1 h. Then, 20 µL of BSTFA with 1% of TMCS were added, vortex-mixed for 5 min, and placed in the oven at 70 °C for 1 h. Finally, 100 µL of heptane containing C18:0 methyl ester (5 µg/mL) as internal standard.

### Metabolic fingerprinting by LC- QTOF-MS analysis

The metabolic analysis was performed using an Agilent 1260 Infinity LC System coupled with Q-TOF 6545 MS system (Agilent Technologies, Palo Alto, CA, USA). 5 µL of the extracted sample was injected onto InfinityLab Poroshell 120 EC-C18 column (2.1 × 150 mm 2.7 µm, Agilent) thermostated at 30 °C. The flow rate of mobile phase (A: Milli-Q water with 0.1% formic acid (v/v), B: acetonitrile with 0.1% formic acid (v/v)) was 0.4 mL/min. The gradient elution program started running at 5% B, increasing to 10% B in 7 min, then increasing to 95% B in 15 min and hold at 95% B for 2 min, ending going back to initials conditions in 1 min and held there for 8 min to allowed column re-equilibrium. The analysis was performed with positive and negative ionization mode using two reference masses in each polarity: *m/z* 121.0509 (C_5_H_4_N_4_) and *m/z* 922.0098 (C_18_H_18_O_6_N_3_P_3_F_24_) in positive mode an *m/z* 112.9856 (C_2_O_2_F_3_(NH_4_)) and *m/z* 1033.9881 (C_18_H_18_O_6_N_3_P_3_F_24_) for negative ionization mode. The system was operated in full scan mode from 100 to 1100 m*/z*. The capillary voltage was set to 3500; the drying gas flow rate was 8 L/min at 325 °C, gas nebulizer 50 psi, fragmentation voltage 175 V and skimmer 65 V and octopole radio frequency voltage (OCT RF Vpp) 750 V. Data were collected in the centroid mode at a scan rate of 1.00 spectrum per second.

### Metabolic fingerprinting by GC-QTOF-MS analysis

Metabolic fingerprinting was performed using an Agilent Technologies 7250 GC/QTOF system (Agilent Technologies, Palo Alto, CA, USA). 2 µL of the derivatized sample was injected onto an HP-5MS UI column (30 m × 0.25 mm × 0.25 µm), using helium as a carrier gas at a constant gas flow of 0,7 mL/min. Injector temperature was set at 280 °C and split ratio to 30:1. The gradient temperature program started at 60 °C, held there for 1 min, and then started to increase to 325 °C with a rate of 10 °C/min. The GC–MS transfer line was set at 280, filament source at 250, and quadrupole temperature at 150. The electron ionization (EI) source was set at 70 eV, and the mass spectrometer was operated in full scan mode from 50 to 600 m*/z* at a scan rate of 5.00 scans/s.

### Quality control samples

Quality control (QC) samples were prepared by mixing equal volumes of extracted samples. To determine the reproducibility and stability of the analytical platforms used, several QC runs were performed before analyzing all cocoa samples until system equilibration was achieved and every five randomized samples.

### Data treatment

All raw data were processed as previously reported by Cala et al^30^. In summary, for LC-QTOF-MS data processing was performed withAgilent MassHunter Profinder B.10.0 Software for deconvolution, alignment, and integration.. GC–MS data processing consisted of a deconvolution step with Agilent MassHunter Unknowns Analysis B.10.00 and metabolite identification using the libraries Fiehn version 2015 and NIST 17. Agilent Mass Profiler Professional B.12.1 software was used for retention time alignment, and then withAgilent MassHunter Quantitative B.10.00 was performed the integration of each metabolite following Agilent guidelines. Both LC–MS and GC–MS data were inspected manually to clean thenoise. Finally, data were filter by presence and reproducibility, maintaining the metabolites present in 100% of the biological replicates in each group and a coefficient of variation in the QC lower than 20%.

### Statistical analysis

To determine statistically significant differences between metabolomics profiles, multivariate (MVA) statistical analyses were performed using SIMCA 16.0 (Umetrics, Umea, Sweden). Principal component analysis (PCA) was applied to evaluate the acquired data quality, verifying that the QC samples were correctly clustered in these models to guarantee the stability of the analytical system. After that, PLS-DA and OPLS-DA models were built to maximize and inspect the differences between study groups and select responsible metabolites for separating the groups. Pareto scaling were used before the statistical analysis. For all platform data, the significant variables were selected by keeping only the variables that fulfilled:1) MVA criteria (variance significant in projection (VIP) > 1.5 with Jack-knife confident interval (JK) not including the zero value from orthogonal partial least-squares discriminant analysis (OPLS-DA) with CV-ANOVA < 0.05) and 2) Change percent > 30%.

### Metabolites identification

Accurate masses of features representing significant differences in class separation identified by all platforms were searched in the following databases: KEGG (http:// genome.jp/keg) and Lipid MAPS (http://lipidmaps.org), METLIN (http://metlin.scripps.edu) using the CEU Mass Mediator tool^31^. Then LC − MS/MS analyses were performed to confirm the metabolite's identity.

## Results and discussion

### A Multiplatform metabolic fingerprinting of fine-flavor cocoa fermentation was obtained through LC-QTOF-MS and GC-QTOF-MS

**Figure 1:**
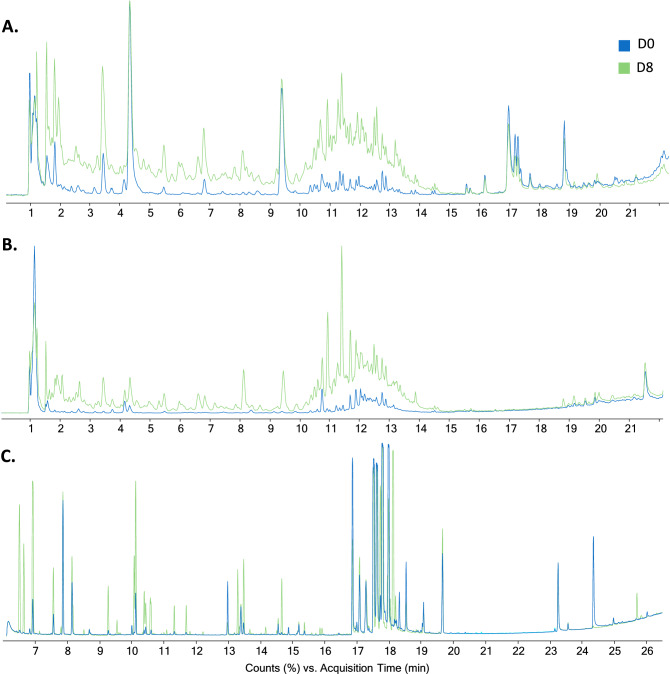
Comparison of total ion chromatogram of metabolic profiles for day 0 (D0) and day 8 (D8) during fermentation of fine-flavor cocoa. (**A**) Metabolic fingerprinting (MF) by LC–MS( +); (**B**) MF by LC–MS(-); (**C**) MF by GC–MS.

**Figure 2:**
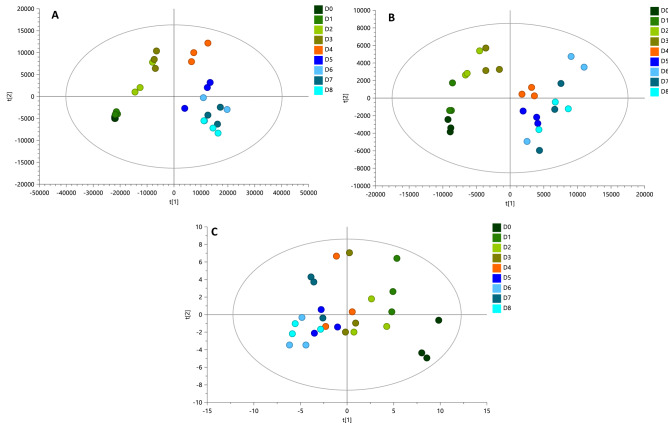
PCA score plots for all fermentation samples. (**A**) MF by LC–MS( +): R^2^(cum): 0.875, Q^2^ (cum): 0.826; (**B**) MF by LC–MS(-): R^2^(cum): 0.876, Q^2^ (cum): 0.77; (**C**) MF by GC–MS: R^2^(cum): 0.724, Q^2^ (cum): 0.229.

We used LC-QTOF-MS and GC-QTOF-MS to elucidate the metabolic activity through the cocoa fermentation process. The total coverage of molecular features from the metabolic fingerprinting (MF) after data processing and filtering that we obtained consisted of 1497, 551, and 71 features by LC-QTOF-MS( +), LC-QTOF-MS(-), GM-and GC-QTOF-MS( +), respectively. Using quality control (QC) samples clustering, we evaluated the performance of the different analytical platforms in unsupervised PCA models (Supplementary Fig. S1). A clear QC grouping was observed in PCA analysis for all analytical platforms, assuring the quality of acquired data and supporting that separating groups is related to biological and not analytical variations. Overall, we observed a significant difference between the metabolic fingerprint of the beginning (day 0) and the end (day 8) of the fermentation (Fig. 1), generally characterized by an increase in the signals of fermentation day 8. For all platforms, the PCA analysis revealed a clear separation between the metabolic fingerprint of day 0 and day 8 of the fermentation and a change in metabolite throughout all fermentation days (Fig. 2). However, the analysis of the metabolic fingerprint for each day revealed only a slight separation between specific days such as day 0 and day 1, day 2 and day 3, and the last three days of fermentation (i.e., day 6, day 7, and day 8), suggesting that these days exhibit a similar metabolic profile. This same behavior has been previously reported in several studies^24,25,28^.

### Two different phases of metabolic activity were detected during spontaneous fermentation of fine-flavor cocoa beans

**Figure 3:**
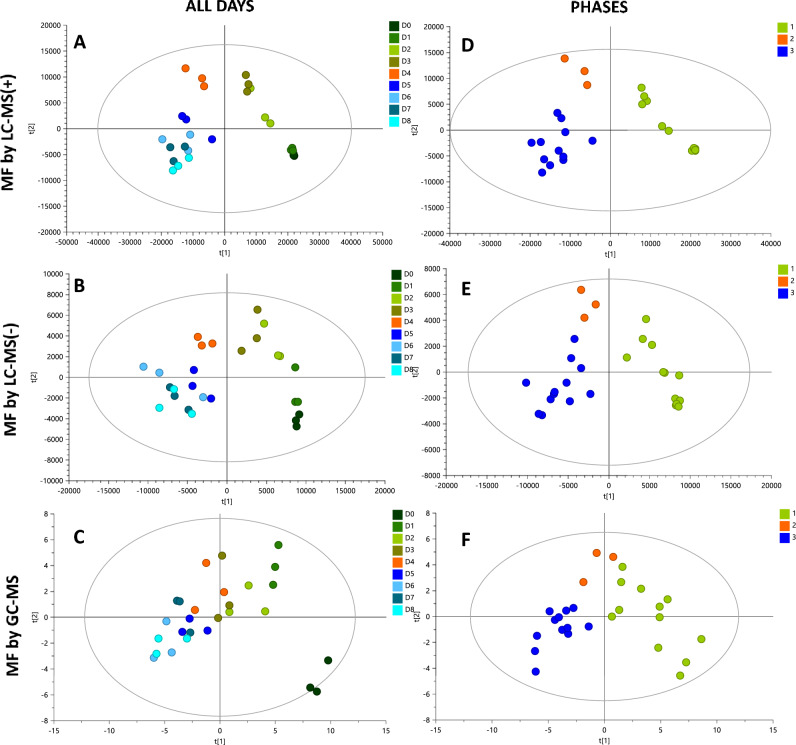
PLS-DA models for all cocoa fermentation samples and the proposed metabolic phases. ALL DAYS: (**A**) MF by LC–MS( +): R^2^(cum): 0.838, Q^2^ (cum): 0.137, cv-anova: 0.995; (**B**) MF by LC–MS(-): R^2^(cum): 0.696, Q^2^ (cum): 0.083, cv-anova: 0.993; (**C**) MF by GC–MS: R^2^(cum): 0.442, Q^2^ (cum): 0.084, cv-anova: 0.951. PHASES: (**D**) MF by LC–MS( +): R^2^(cum): 0.907, Q^2^ (cum): 0.899, cv-anova: 6.296e−07; (**E**) MF by LC–MS(-): R^2^(cum): 0.903, Q^2^ (cum): 0.804, cv-anova: 0.00053; (**F**) MF by GC–MS: R^2^(cum): 0.415, Q^2^ (cum): 0.45, cv-anova: 0.0188.

**Figure 4:**
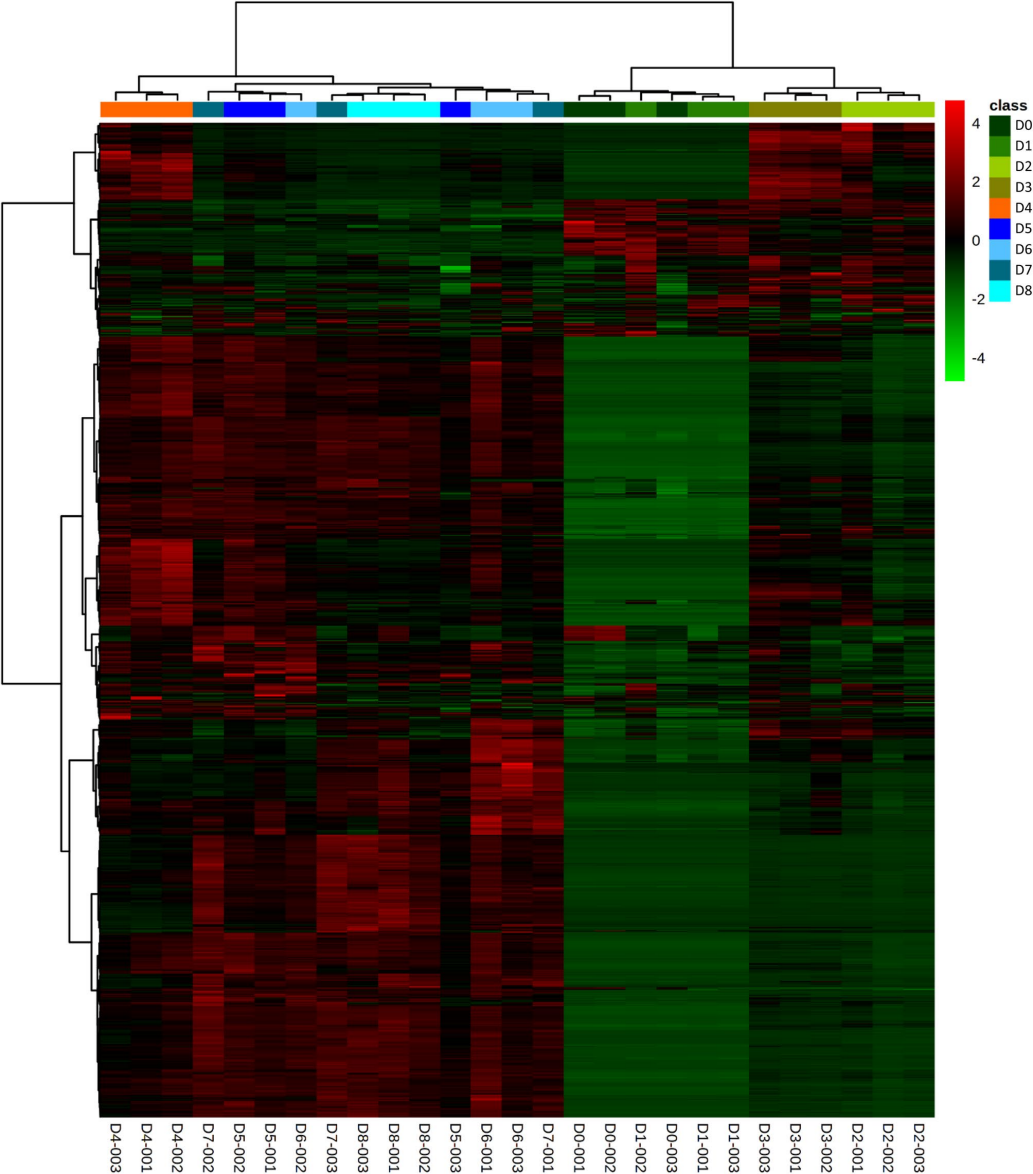
Heat map analysis of metabolite features detected in all analytical platforms for all fermentation days (MetaboAnalyst 5.0^68^). The color spectrum ranging from red to green indicates the range of high to low signal intensities for each metabolite.

**Figure 5:**
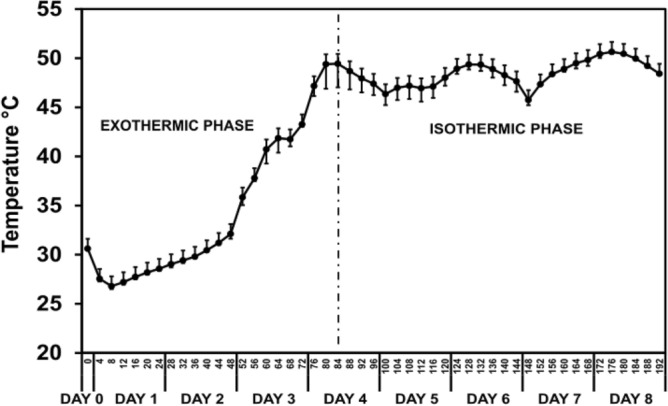
Temperature dynamic during the fermentation of fine-flavor cocoa. The dotted line divides cocoa fermentation temperature dynamics into two phases.

PLS-DA models were built to explore and maximize the differences in metabolic fingerprint throughout the fermentation process (Fig. 3A–C). The PLS-DA models showed little discrimination in the metabolic fingerprint of the fermentation process on each day in all analytical platforms; however, in the PLS-DA score plots, it is possible to observe three clusters of fermentation days corresponding to D0-D3, D4, and D5-D8. Using this approach, additional PLS-DA models were built to explore the differences between these three groups (Fig. 3D–F). A clear separation was observed in the score plots for all PLS-DA models between these groups with high quality, proven by significant variance values explained (R^2^), variance predicted (Q^2^), and CV-ANOVA. These results suggest that the most prominent metabolites changes cluster in two phases (D0 to D3 and D5 to D8) during the fermentation process of cocoa beans. To further explore the trends in metabolites modifications along fermentation days, a heatmap was built for all metabolite features detected in all analytical platforms using MetaboAnalyst 5.0 (Fig. 4). The groups (days) in the diagram were allocated using a hierarchical clustering algorithm, joining them by similarity, as indicates the dendrogram on the top of the figure. This heatmap shows two similar metabolic fingerprint with differential alteration of a significant number of metabolites between them. The first metabolic fingerprint goes from day 0 to day 3 and the second one from day fifth on, supporting the presence of two major metabolic phases during the fermentation of fine-flavor cocoa. Interestingly, these two phases are concomitant with the temperature profile in the cocoa mass through the entire process (Fig. 5).

### The temperature of the fermenting mass is a crucial parameter during spontaneous fermentation of fine-flavor cocoa

Our data revealed two different temperature phases during spontaneous cocoa fermentation (Fig. 5) concomitant to the two phases of differential metabolic activity previously described (Figs. 3 and 4). The first phase is essentially exothermic. During this phase, the temperature of the fermenting mass significantly increased from 26.8 ± 0.3 °C to 49.4 ± 2.5 °C. Contrary, the second phase (from the fifth day on) is primarily isothermic. The temperature during second phase remained between 45 and 50 °C until the fermentation ended, a variation 81% lower compared with the exothermic phase. This kind of temperature profile was reported in different cocoa fermentations worldwide, with different cocoa varieties, fermentation methods, and weather conditions^20,21,32,33^.

Temperature rise, usually more than 25 °C over the initial temperature of cocoa mass, could be associated with exothermic reactions (e.g., ethanol and weak acids production) from the first days of fermentation^10,34,35^. A maximum temperature increase rate was observed on fermentation day 4, which corresponds with the first turning of the fermenting mass to allow oxygenation. Thus, oxygen availably could partially explain the increase in temperature as a substantial increase in oxygen concentration may lead to a dramatic rise in exothermic oxidation reactions^36,37^. The temperature over 45 °C is a critical control point to determine the quality of the process, independently of the fermentation method, the region, the cocoa type, or the required fermentation time^20,33,37–39^.

As mentioned before, from the fifth day on, the temperature experienced minor changes and remained between 45–50 °C until fermentation ends. During this final phase, most microbial populations, and eventually its associated biochemical activity, decline as previously observed^13,14,40^. The relative abundance of microbial groups as yeast and LAB significantly decreases the last days of cocoa fermentation^13,15,40^. This drop is caused by a considerable decrease of fermentable sugars in the medium (e.g., glucose, fructose)^10,11^. Also, temperature plays a relevant role in controlling microbial populations considering that LAB and yeast growth decreases dramatically over 40 °C^41,42^. However, the elevated presence of lactic acid and oxygen prompts the growth of AAB that can resist higher temperature, producing acetic acid (an exothermic process) and releasing heat to the medium at a considerably lower rate but sufficient to maintain cocoa mass temperature relatively high, with a maximum variation of 5 °C^35,43^.

The temperature rise has a critical impact on flavor development during cocoa fermentation, as it guarantees the formation of molecules responsible for a high-quality sensorial profile^44^. The production of these compounds evidence the influence of temperature in metabolic activity across cocoa fermentation and the quality of the cocoa^10,44^. A high temperature causes a significant diffusion of acetic acid, lactic acid, and ethanol into the beans promoting the degradation of flavonoids, reducing the bitterness and astringency of the cocoa^1,39^. Also, it changes more than 25 °C during the fermentation^10,37,44^, stimulating a wide range of biochemical reactions and the growth of specific microorganisms in each increasing stage. These microorganisms carried out most of the transformations occurring in cocoa fermentation^37,45^. To achieve a high-quality sensorial profile, a proper succession of some specific microorganism genera is crucial. The development of this microbial progression depends on substrates present in the media and temperature^39^.

Additionally, temperature plays a crucial role in proteolysis. The temperature reaches more than 45 °C during cocoa fermentation. This high temperature causes the activation of some native enzymes, and the protein heat denaturation produces a significant amount of peptides and amino acids^25,46^. These molecules are flavor precursors associated with the development of fruits and nutty notes in cocoa, qualities quite appreciated in the international markets^10,47,48^.

### The variation in dominant groups of metabolites diverge between the two phases of differential metabolic activity

**Figure 6:**
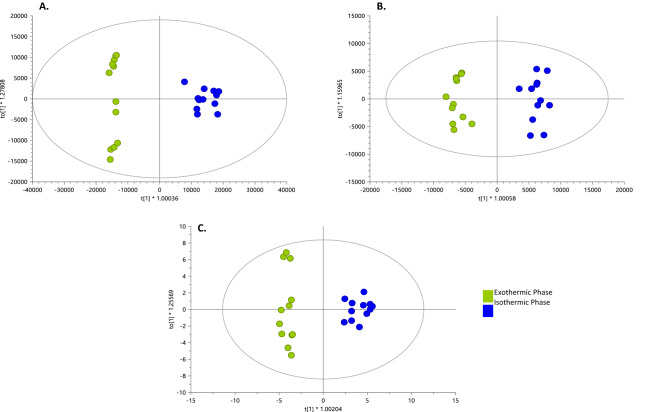
OPLS-DA models for two phases. (**A**) MF by LC–MS( +): R^2^_(cum)_: 0.804, Q^2^_(cum)_: 0.962, cv-anova: 3.58e−13; (**B**) MF by LC–MS(-): R^2^_(cum)_: 0.739, Q^2^_(cum)_: 0.96, cv-anova: 5.67e−07; (**C**) MF by GC–MS: R^2^_(cum)_: 0.702, Q^2^_(cum)_: 0.864, cv-anova: 5.86e−12.

We analyzed the effect of the temperature dynamic in the metabolic profile of cocoa fermentation using a multiplatform metabolomics analysis. This approach allowed us to propose a new paradigm of the phases of cocoa fermentation based on the global metabolic activity instead of the variation in only a few metabolites. We propose two phases of cocoa fermentation based on the temperature dynamic: an exothermic phase from day 0 to day 3 and an isothermic phase from day 5 to day 8. Once these phases were established, univariate (*p*-value < 0.05 from hypothesis testing) and multivariate (OPLS-DA models) analyses were performed to select the differential metabolites between each phase. For all analytical platforms, the OPLS-DA analysis allowed modeling the differences between the two phases with statistically significant values for R^2^, Q^2^, CV-ANOVA (Fig. 6). As a result, 44, 65, 20 differential metabolites were identified in LC-QTOF-MS( +), LC-QTOF-MS(-), and GC-QTOF-MS( +), respectively.

Table 1 shows metabolites altered between the exothermic and isothermic phases of cocoa fermentation. The biggest group of metabolites differentially expressed correspond to amino acids, dipeptides, and tripeptides. The concentration of these 23 molecules increased in the isothermic phase. The formation of amino acids and peptides during cocoa fermentation is due to protein hydrolysis and denaturation processes. Protein content (e.g., albumin, prolamin, globulin, and glutein) represents 10 –15% of dry unfermented cocoa beans^46,47^ and is significantly lower for fermented cocoa beans^38,47,49^. The drop in protein content, usually above 60%, is caused by proteolysis during fermentation that involves two groups of native cocoa proteases: endoproteases and carboxypeptidases^38,47,49^. The optimal temperature for these enzymes is 45–50°C^50,51^, precisely the same temperature range of the fermentation the isothermic phase. Also, this temperature itself is accountable for protein denaturation, breaking them down into peptides or even amino acids^25,49^. This protein degradation linked to enzymatic hydrolysis and denaturation by heat in cocoa fermentation has been widely documented in several studies^25,46–49^.

**Table 1:** Metabolites with statistical significance in the two phases of the fermentation process identified by metabolic fingerprinting using LC-QTOF-MS( ±) and GC-QTOF-MS( +).

Compound name	Molecular Formula	Molecular weight (DB) g/mol	RT (min)	Mass error (ppm)	Analytical platform	DET	CON	CV in QC (%)	Fold Change	VIP Value	*p-*value with FDR
***Amino acids, peptides, and derivatives***											
Alanine	C_3_H_7_NO_2_	89.0476	7.553	–	GC-QTOF-MS	EI	Identified	1.85	1.3	1.098	0.0034
Aspartic acid	C_4_H_7_NO_4_	133.0375	8.11	7	LC-QTOF-MS	ESI-	Putative	1.25	6.6	2.650	0.00019
Glutamic acid	C_5_H_9_NO_4_	47.1293	14.55	–	GC-QTOF-MS	EI	Identified	2.9	2.1	1.34247	–
Glutamine	C_5_H_10_N_2_O_3_	146.0691	1.81	3	LC-QTOF-MS GC-QTOF-MS	ESI + EI	Putative Identified	1.25	5.3	2.336	7.22 E−05
Leucine	C_6_H_13_NO_2_	131.0946	2.08	5	LC-QTOF-MS	ESI-	Putative	2.57	3.7	1.524	9.58 E−05
Phenylalanine	C_9_H_11_NO_2_	165.079	3.44	2	LC-QTOF-MS GC-QTOF-MS	ESI + EI	Putative Identified	2.42	3.3	1.537	9.58 E−05
Proline	C_5_H_9_NO_2_	115.0633	2.69	5	LC-QTOF-MS GC-QTOF-MS	ESI + EI	Putative Identified	13.58	3.3	1.790	0.00011
Serine	C_3_H_7_NO_3_	105.0926	11.32	–	GC-QTOF-MS	EI	Identified	3.3	5.1	1.61403	–
Threonine	C_4_H_9_NO_3_	119.1192	11.69	–	GC-QTOF-MS	EI	Identified	1.4	5.4	1.6294	–
Tyramine	C_8_H_11_NO	137.0841	2.81	2	LC-QTOF-MS	ESI +	Putative	2.88	4.6	1.839	7.22 E−05
Tyrosine	C_9_H_11_NO_3_	181.0739	1.83	3	LC-QTOF-MS	ESI-	Putative	2.57	2.5	1.424	8.61 E−07
Caffeoyl aspartic acid	C_13_H_13_NO_7_	295.0692	8.12	2	LC-QTOF-MS	ESI-	Putative	1.78	8.8	3.820	0.00019
AlanylProline	C_8_H_14_N_2_O_3_	186.1004	2.26	2	LC-QTOF-MS	ESI +	Putative	7.00	7.0	2.000	9.59 E−09
ArginylProline	C_11_H_21_N_5_O_3_	271.1644	1.94	6	LC-QTOF-MS	ESI +	Putative	4.03	28.8	1.627	7.22 E−05
Glutamylglutamine	C_10_H_17_N_3_O_6_	275.1117	1.9	0	LC-QTOF-MS	ESI-	Putative	1.99	11.7	1.161	9.10 E−05
Glutamylleucine	C_11_H_20_N_2_O_5_	260.1372	2.07	2	LC-QTOF-MS	ESI ±	MS/MS	1.42	6.1	1.744	7.22 E−05
LeucylThreonine	C_10_H_20_N_2_O_4_	232.1423	1.72	2	LC-QTOF-MS	ESI +	Putative	2.44	2.5	1.561	0.00021
ProlylLysine	C_11_H_21_N_3_O_3_	243.1583	2.52	2	LC-QTOF-MS	ESI-	Putative	3.17	6.4	1.794	9.10 E−05
ProlylSerine	C_8_H_14_N_2_O_4_	202.0954	2.07	2	LC-QTOF-MS	ESI +	Putative	1.90	26.0	2.170	7.22 E−05
Tripeptide 1	C_17_H_29_N_7_O_5_	411.223	1.64	7	LC-QTOF-MS	ESI ±	Putative	1.80	10.0	2.162	7.22 E−05
Tripeptide 2	C_13_H_25_N_3_O_5_	303.1794	1.88	0	LC-QTOF-MS	ESI ±	Putative	1.61	2.6	1.649	0.00086
Tripeptide 3	C_16_H_29_N_5_O_7_	403.2067	1.93	1	LC-QTOF-MS	ESI ±	Putative	1.52	12.6	2.848	7.22 E−05
Tripeptide 4	C_17_H_22_N_4_O_8_	410.1438	3.28	0	LC-QTOF-MS	ESI-	Putative	3.43	9.7	1.052	9.10 E−05
***Organic acids***											
Avenanthramide 2	C_18_H_17_NO_7_	359.1005	11.98	2	LC-QTOF-MS	ESI-	Putative	3.38	14.5	1.187	9.58 E−05
Benzoic acid	C_7_H_6_O_2_	122.0368	11.39	5	LC-QTOF-MS	ESI +	Putative	1.42	12.6	2.364	0.00013
Caffeic acid	C_9_H_8_O_4_	180.0423	8.1	2	LC-QTOF-MS	ESI +	MS/MS	0.86	4.8	4.299	0.00016
Coumaric acid	C_9_H_8_O_3_	164.0473	11.39	4	LC-QTOF-MS	ESI +	MS/MS	2.07	10.0	1.888	0.00013
Oxoglutaric acid	C_5_H_6_O_5_	146.0215	1.58	5	LC-QTOF-MS	ESI-	Putative	6.36	1.5	1.079	3.99 E−05
Salicylic acid	C_7_H_6_O_3_	138.0317	11.4	3	LC-QTOF-MS	ESI-	Putative	2.08	13.8	1.691	0.00015
Sesamol	C_7_H_6_O_3_	138.0317	11.39	4	LC-QTOF-MS	ESI ±	Putative	1.28	10.3	3.352	0.00013
Tetrahydroxychalcone 4'-*O*-(2''-*O*-acetyl-6''-*O*-cinnamoyl)glucoside	C_32_H_30_O_12_	606.1737	12.64	1	LC-QTOF-MS	ESI-	Putative	3.81	14.1	1.434	9.10 E−05
Tumonoic Acid H	C_26_H_45_NO_7_	483.3196	4.62	5	LC-QTOF-MS	ESI +	Putative	3.06	12.2	2.671	7.20 E−05
***Carbonyl compounds***											
Acetophenone	C_8_H_8_O	120.0575	12.26	6	LC-QTOF-MS	ESI-	Putative	2.51	6.8	1.271	0.0011
Acetol	C_3_H_6_O_2_	74.0785	13.29		GC-QTOF-MS	EI	Identified	1.7	5.6	1.23759	0.0001
Hydroxyacetophenone	C_8_H_8_O_2_	136.0524	8.11	6	LC-QTOF-MS	ESI-	Putative	2.04	6.1	1.877	0.00015
***Carbohydrates***											
Anhydrofructose	C_6_H_10_O_5_	162.0528	1.16	4	LC-QTOF-MS	ESI +	Putative	6.46	0.2	1.540	7.22 E−05
Arabitol//Ribitol	C_5_H_12_O_5_	152.0685	15.59	–	GC-QTOF-MS	EI	Identified	0.0	2.0	1.04962	0.00016
Decarboxybetanin	C_23_H_27_N_2_O_11_	507.1615	4.42	4	LC-QTOF-MS	ESI-	Putative	1.63	15.0	1.158	9.10 E−05
Dihydrocaffeic acid 3-*O*-glucuronide	C_15_H_18_O_10_	358.09	1.14	2	LC-QTOF-MS	ESI +	MS/MS	2.33	0.3	2.240	5.04 E−06
Dihydroxyphenyl 1-*O*-(6-*O*-galloyl-beta-D-glucopyranoside)	C_19_H_20_O_12_	440.0955	1.14	5	LC-QTOF-MS	ESI-	Putative	1.39	0.3	1.271	0.00015
Gluconic acid	C_6_H_12_O_7_	196.0583	1.13	6	LC-QTOF-MS	ESI-	Putative	1.27	0.7	2.212	–
Glucose //Galactose	C_6_H_12_O_6_	180.0634	17.61	–	GC-QTOF-MS	EI	Identified	2.6	2.1	1.46337	–
Maltose	C_12_H_22_O_11_	342.2965	24.86	–	GC-QTOF-MS	EI	Identified	4.7	0.0	1.07032	–
Mannitol	C_6_H_14_O_6_	182.1718	17.8	–	GC-QTOF-MS	EI	Identified	3.8	0.8	–	0.0178867
Raffinose	C_18_H_32_O_16_	504.169	1.13	1	LC-QTOF-MS	ESI-	Putative	1.70	0.1	2.429	1.22 E−05
Ribose // Lyxose // Arabinose	C_5_H_10_O_5_	150.0528	15.1	–	GC-QTOF-MS	EI	Identified	1.4	1.3	1.17136	–
Sorbitol	C_6_H_14_O_6_	182.1718	17.86	-	GC-QTOF-MS	EI	Identified	3.5	0.8	1.2679	–
Sorbosone	C_6_H_10_O_6_	178.0477	1.21	6	LC-QTOF-MS	ESI-	Putative	2.87	0.5	1.040	0.00019
Stachyose // Maltotetraose	C_24_H_42_O_21_	666.2219	1.1	0	LC-QTOF-MS	ESI-	Putative	1.68	0.2	2.581	1.09 E−07
***Phenols***											
Catechol	C_6_H_6_O_2_	110.1106	10.59		GC-QTOF-MS	EI	Identified	2.8	1.5	1.22319	0.011
Dopamine	C_8_H_11_NO_2_	153.079	10.11	3	LC-QTOF-MS	ESI +	Putative	3.11	6.1	1.570	7.22 E−05
Methylcatechol	C_7_H_8_O_2_	124.0524	11.4	6	LC-QTOF-MS	ESI-	Putative	2.31	11.9	1.249	0.00015
Phenol	C_6_H_6_O	94.0419	3.42	8	LC-QTOF-MS	ESI +	Putative	2.23	2.5	2.902	8.70 E−05
Pyrocatechol	C_6_H_6_O_2_	110.0368	11.4	6	LC-QTOF-MS	ESI-	Putative	2.88	12.3	1.234	0.00015
Vanillin	C_8_H_8_O_3_	152.0473	8.1	3	LC-QTOF-MS	ESI +	MS/MS	1.29	5.3	2.567	0.00016
Vanillin isobutyrate	C_12_H_14_O_4_	222.0892	11.4	3	LC-QTOF-MS	ESI-	Putative	1.44	12.7	1.213	0.00015
***Indoles and derivatives***											
Indolamine	C_8_H_8_N_2_	132.0687	8.12	3	LC-QTOF-MS	ESI-	Putative	8.33	4.7	1.840	0.00019
Indoleacrylic acid	C_11_H_9_NO_2_	187.0633	10.2	3	LC-QTOF-MS	ESI +	Putative	1.93	8.5	1.808	7.22 E−05
***Phenylpropanoids***											
Schizandrin	C_24_H_32_O_7_	432.2148	10.91	7	LC-QTOF-MS	ESI ±	Putative	1.18	6.8	2.963	7.20 E−05
Sinapine	C_16_H_24_NO_5_	310.1654	6.58	3	LC-QTOF-MS	ESI-	Putative	1.93	15.5	1.195	9.10 E−05
***Pyridine nucleotides***											
Nicotinamide ribotide	C_11_H_15_N_2_O_8_P	334.0566	10.39	8	LC-QTOF-MS	ESI-	Putative	1.55	10.1	1.582	0.00024
***Coumarins***											
Coumarin	C_9_H_6_O_2_	146.0368	10.47	4	LC-QTOF-MS	ESI +	MS/MS	1.56	1.7	1.756	0.011
Methylcoumarin	C_10_H_8_O_2_	160.0524	11.4	4	LC-QTOF-MS	ESI-	Putative	1.38	12.7	1.293	0.00015
Marmesin	C_14_H_14_O_4_	246.0892	9.44	2	LC-QTOF-MS	ESI-	Putative	5.47	16.6	1.452	9.10 E−05
Occidentoside	C_36_H_32_O_15_	704.1741	1.09	6	LC-QTOF-MS	ESI +	MS/MS	1.35	0.3	2.482	1.19 E−07
***Flavonoids***											
Alkannin	C_16_H_16_O_5_	288.0998	12.48	1	LC-QTOF-MS	ESI +	Putative	3.67	3.0	1.649	0.0022
Arecatannin A2	C_60_H_50_O_24_	1154.2692	12.09	2	LC-QTOF-MS	ESI-	Putative	2.14	0.7	1.224	0.044
Arecatannin A3	C_75_H_62_O_30_	1442.3326	12.03	2	LC-QTOF-MS	ESI-	Putative	2.43	0.7	1.576	0.021
A-type procyanidin dimer	C_30_H_24_O_12_	576.1268	13.86	0	LC-QTOF-MS	ESI-	Putative	1.56	1.9	1.050	0.0045
A-type procyanidin dimer	C_30_H_24_O_12_	576.1268	12.48	1	LC-QTOF-MS	ESI +	Putative	1.53	3.1	1.635	0.00086
B-type procyanidin dimer	C_30_H_26_O_12_	578.1424	10.93	0	LC-QTOF-MS	ESI-	Putative	1.96	4.2	5.049	0.00081
B-type procyanidin dimer	C_30_H_26_O_12_	578.1424	11.15	0	LC-QTOF-MS	ESI-	Putative	2.17	4.9	2.153	0.00054
B-type procyanidin dimer	C_30_H_26_O_12_	578.1424	12.48	1	LC-QTOF-MS	ESI ±	MS/MS	1.29	3.1	1.586	0.0011
C-type procyanidin trimer	C_45_H_38_O_18_	866.2058	10.9	4	LC-QTOF-MS	ESI-	Putative	1.49	2.5	2.042	0.0031
Butein -arabinosyl-galactoside	C_26_H_30_O_14_	566.1636	1.12	9	LC-QTOF-MS	ESI-	Putative	1.75	0.3	1.318	4.91 E−08
Caffeoylpelargonidin 5-glucoside	C_30_H_27_O_13_	595.1452	12.09	2	LC-QTOF-MS	ESI-	Putative	1.41	0.8	1.146	–
Catechin	C_15_H_14_O_6_	290.079	11.39	2	LC-QTOF-MS	ESI ±	MS/MS	2.03	12.5	2.733	0.00013
Epicatechin	C_15_H_14_O_6_	290.079	12.48	4	LC-QTOF-MS GC-QTOF-MS	ESI- EI	Putative Identified	1.76	3.1	1.110	0.00012
Epicatechin 3-glucoside	C_21_H_24_O_11_	452.1319	8.66	1	LC-QTOF-MS	ESI-	Putative	3.16	5.9	1.176	0.00029
Epigallocatechin 3-*O*-(4-hydroxybenzoate)	C_22_H_18_O_9_	426.0951	10.93	2	LC-QTOF-MS	ESI-	Putative	2.06	4.3	1.824	0.00081
Isoflavonoid *O*-glycosides	C_28_H_34_O_12_	562.205	11.26	4	LC-QTOF-MS	ESI-	Putative	1.03	7.2	1.460	9.10 E−05
Kaempferol -diacetyl-coumarylrhamnoside	C_34_H_30_O_14_	662.1636	12.74	2	LC-QTOF-MS	ESI-	Putative	3.25	2.4	1.324	0.0017
Kaempferol-caffeoylglucosyl-rhamnoside	C_36_H_36_O_18_	756.1902	12.59	1	LC-QTOF-MS	ESI-	Putative	2.33	2.2	1.329	0.0017
Viscumneoside V	C_32_H_40_O_19_	728.2164	1.11	6	LC-QTOF-MS	ESI-	Putative	2.02	0.4	1.378	1.36 E−08
***Purines and purine derivatives***											
Hydroxyadenine // Guanine	C_5_H_5_N_5_O	151.0494	1.96	3	LC-QTOF-MS	ESI +	MS/MS	1.08	3.1	3.264	7.22 E−05
Xanthine	C_5_H_4_N_4_O_2_	152.0334	1.96	2	LC-QTOF-MS	ESI +	Putative	1.66	3.7	1.660	7.22 E−05
***Organoheterocyclic compound***											
Cytosine	C_4_H_5_N_3_O	111.0433	1.21	6	LC-QTOF-MS	ESI +	MS/MS	1.74	3.9	2.042	2.00 E−12
Maltol	C_6_H_6_O_3_	126.0317	11.4	4	LC-QTOF-MS	ESI-	Putative	7.79	9.7	1.239	0.00015
***Terpenoids***											
Cedrol	C_15_H_26_O	222.1984	10.59	4	LC-QTOF-MS	ESI +	Putative	1.83	7.7	1.941	7.22 E−05
Irone	C_14_H_22_O	206.1671	10.69	5	LC-QTOF-MS	ESI +	Putative	1.58	35.7	3.768	7.22 E−05
Salannin	C_34_H_44_O_9_	596.2985	18.8	5	LC-QTOF-MS	ESI-	Putative	4.87	4.0	1.836	9.58 E−05
***Glycosides***											
Methylthiooctyl-desulfoglucosinolate	C_16_H_31_NO_6_S_2_	397.1593	4.15	0	LC-QTOF-MS	ESI-	Putative	2.22	1.2	1.029	0.0082
***Phenylpropanoids and polyketides***											
Resveratrol 3-sulfate	C_14_H_12_O_6_S	308.0355	11.94	0	LC-QTOF-MS	ESI-	Putative	1.34	5.1	1.150	0.00066
Oleandolide	C_20_H_34_O_7_	386.4798	4.22	5	LC-QTOF-MS	ESI +	Putative	1.38	32.8	2.074	7.20 E−05
***Fatty acyls***											
Heptadecanoic acid	C_17_H_34_O_2_	268.4348	19.81		GC-QTOF-MS	EI	Identified	2.7	1.3	–	0.00022
Hydroxyandrostane-3-glucuronide	C_25_H_40_O_9_	484.2672	2.54	3	LC-QTOF-MS	ESI ±	Putative	0.76	5.4	3.033	7.22 E−05
hydroxy-tetradecenoic acid	C_14_H_26_O_3_	242.1882	11.87	9	LC-QTOF-MS	ESI-	Putative	3.44	9.4	1.235	9.58 E−05
Keto-decanoylcarnitine	C_17_H_31_NO_5_	329.2202	11.6	7	LC-QTOF-MS	ESI-	Putative	1.94	3.9	1.062	0.00021
Linalool xylosyl-glucoside	C_21_H_36_O_10_	448.2308	5.96	4	LC-QTOF-MS	ESI ±	Putative	1.85	57.7	3.051	7.20 E−05
Methylbutanoyl)-6-apiosylglucose	C_16_H_28_O_11_	396.1632	4.31	1	LC-QTOF-MS	ESI-	Putative	6.42	1.8	1.053	0.00049
Methylmalate	C_5_H_8_O_5_	148.0372	1.15	7	LC-QTOF-MS	ESI-	Putative	1.62	0.5	1.532	0.00015
Myristic acid	C_14_H_28_O_2_	228.3709	16.94	–	GC-QTOF-MS	EI	Identified	2.5	1.3	–	0.0051
Oleic acid	C_18_H_34_O_2_	282.4614	20.47	–	GC-QTOF-MS	EI	Identified	12.0	1.3	1.03152	-
Stearic acid	C_18_H_36_O_2_	284.4772	20.68	–	GC-QTOF-MS	EI	Identified	2.7	1.2	1.24361	-
***Lipids***											
Deoxyestradiol	C_18_H_24_O	256.1827	11.87	4	LC-QTOF-MS	ESI ±	Putative	1.90	11.6	2.035	7.22 E−05
LPC(14:0)	C_22_H_44_NO_8_P	481.2805	13.35	4	LC-QTOF-MS	ESI-	Putative	1.05	17.3	1.689	9.10 E−05
LPC(18:2)	C_26_H_50_NO_7_P	519.3325	19.92	1	LC-QTOF-MS	ESI +	MS/MS	5.83	5.6	3.004	0.00011
LPC(O-18:1)	C_26_H_52_NO_7_P	521.3481	21.21	1	LC-QTOF-MS	ESI +	MS/MS	5.20	4.2	2.800	0.00031
Palmitoyl 3-carbacyclic Phosphatidic Acid	C_20_H_39_O_5_P	390.2535	10.19	7	LC-QTOF-MS	ESI +	Putative	3.58	16.1	2.141	7.22 E−05
PI(18:1)	C_27_H_51_O_12_P	598.3118	20	2	LC-QTOF-MS	ESI-	Putative	16.18	2.0	1.061	0.0072
PS(21:0)	C_27_H_54_NO_9_P	567.3536	21.26	3	LC-QTOF-MS	ESI-	Putative	15.59	2.7	1.082	0.0012
Sphinganine	C_18_H_39_NO_2_	301.2981	18.83	2	LC-QTOF-MS	ESI +	MS/MS	3.08	0.6	1.653	0.00016
Sulfoglycolithocholate	C_26_H_42_NO_7_S	512.2682	6.05	5	LC-QTOF-MS	ESI ±	Putative	1.85	35.0	2.272	7.22E−05

^a^DET: detection mode; ^b^CV, coefficient of variation of metabolites in QC samples; ^c^VIP, Variable importance in projection; ^d^CON: Confirmation; ^e^ *p*-value obtained after Benjamini–Hochberg correction test.

*LC* Liquid Chromatography, *QTOF-MS* quadrupole time-of-flight mass spectrometry.

Peptides and amino acids are highly relevant for the nutraceutical and sensorial properties of fine-flavor cocoa. Several cocoa peptides have been associated with bioactive properties such as antioxidant, antihypertensive, and antimicrobial^52^, although further research is still required. On the other hand, peptides and amino acids are precursors of pyrazines produced during drying and roasting through Maillard reactions^5,49^. These pyrazines are responsible for fine-flavor cocoa sensorial notes as fruity, floral, and cocoa^5,53^. In this regard, proteolysis could explain why cocoa fermentation in which temperature is consistently below 45 °C, the obtained chocolate usually has a poor sensorial profile.

Flavonoids are the second largest group of molecules differentially expressed with 18 compounds. In the isothermic phase, we observed a rise of different flavonoids such as dimers and trimers of procyanidins, polyphenol glycosides, and rhamnose-containing polyphenols. Also, our data show a decrease in some arecatannin types and butein and pelargonidin derivates. Although the overall content of flavonoids is expected to decrease throughout fermentation, these degradation processes can cause the emergence of polyphenolic dimers and trimers as well as polyphenolic acids as caffeic acid, benzoic acid, and coumaric acid^54,55^. For instance, oxidation and polyphenol oxidase hydrolysis of complex flavonoids such as anthocyanins, procyanidins, epigallocatechin, and kaempferol results in the formation of dimers and trimers of procyanidins and rhamnose-containing polyphenols^54,55^. However, these derivates tend to have lower bioactivity, leading to a significant drop in the bioactive properties of cocoa throughout fermentation, as previously reported^8,38,56^. Polyphenol degradation and eventual derivates formation can be more pronounced at high temperatures^56,57^.

Contrasting that, carbohydrates are the compound group with a higher number of decreasing concentration metabolites from the exothermic to isothermic phase. These metabolites are a primary substrate for many yeasts and bacteria, eventually generating a rise in different organic acids beyond traditional ones (e.g., lactic acid, acetic acid) like benzoic acid, caffeic acid, coumaric acid, oxoglutaric acid, and salicylic acid, as our data revealed. The carbohydrates transformation into organic acids is more intense during the exothermic than isothermic phase due to temperatures under 40 °C and high content of pulp rich in carbohydrates^11,21,39^. These conditions facilitate yeast and bacteria growth as *Saccharomyces*, *Candida*, *Mallasezia*, *Hanseniospora*, *Lactobacillus*, and *Bacillus* produce organic acids as part of their central and secondary metabolism^13,35,36,40,58^.

Other small metabolite groups as phenols, coumarins, terpenoids, fatty acyls, and lipids were also found to varybetween the two phases. These compounds are likely a byproduct of the degradation of complex molecules as polyphenols using the central and secondary metabolism of many microorganisms associated with cocoa fermentations^13,40,54^. For instance, the yeast of genus such as *Mallasezia*, previously reported in cocoa fermentation, is highly active in the metabolism of lipids and fatty acyls^13^. However, as the link between microorganisms-metabolites is still not elucidated, further research is required to dissect the connection between microbial populations and altered metabolites during cocoa fermentations.

### Changes in the concentration of relevant metabolites (i.e., flavor precursors and bioactive compounds) were observed during spontaneous fermentation of fine-flavor cocoa

**Figure 7:**
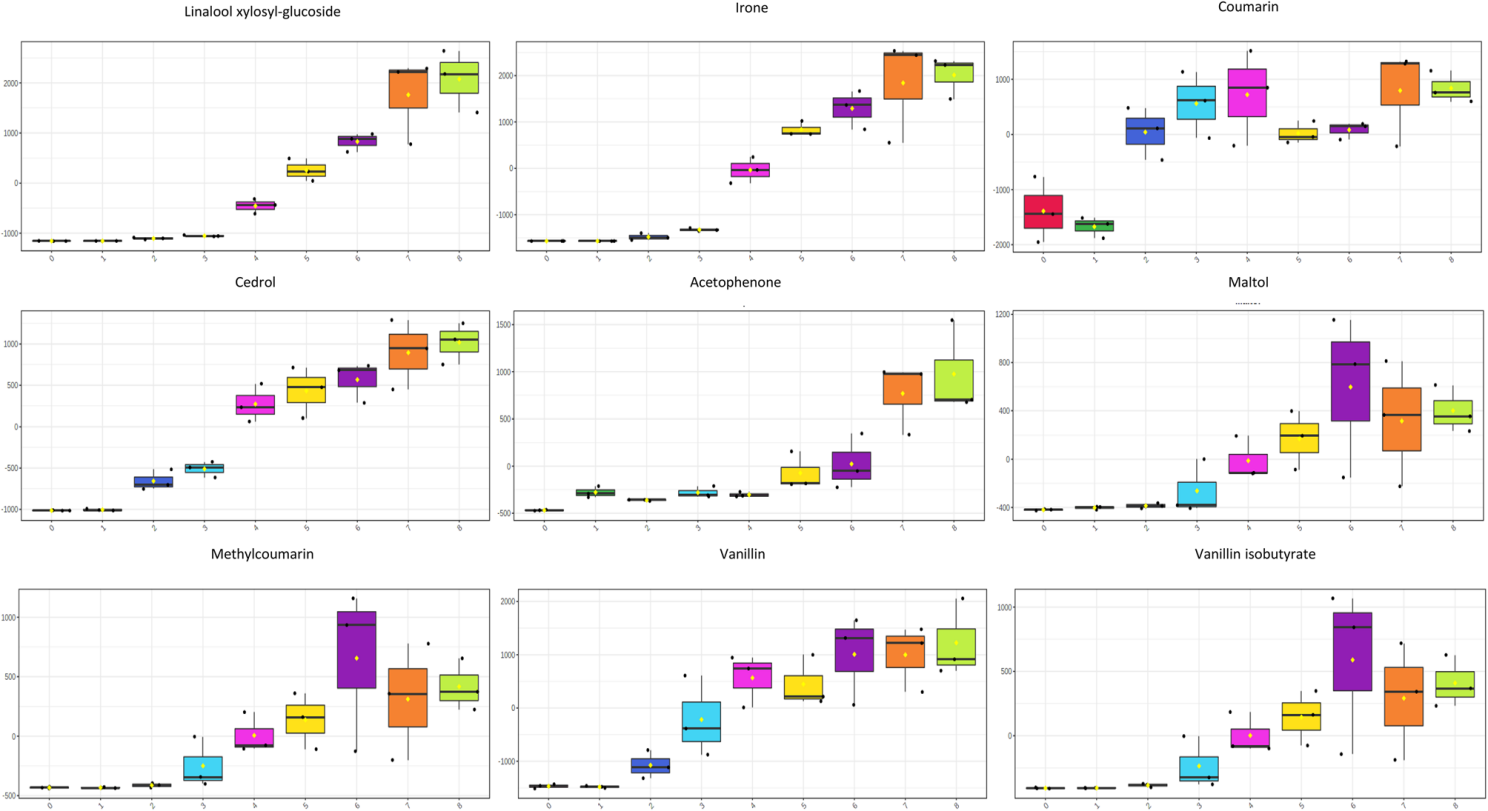
Box plots of metabolites linked with fine-flavor cocoa sensorial notes significantly differ throughout fermentation days (one-way ANOVA correcting for false discovery rate, FDR). Linalool xylosyl-glucoside (fruity, floral), irone (floral), coumaric acid (floral), cedrol (floral, woody,sweet), acetophenone (floral), maltol (caramel, fruity), methylcoumarin (fruity,floral), vanillin (vanilla, chocolate), and vanillin isobutyrate (caramel, chocolate, fruity).

**Figure 8:**
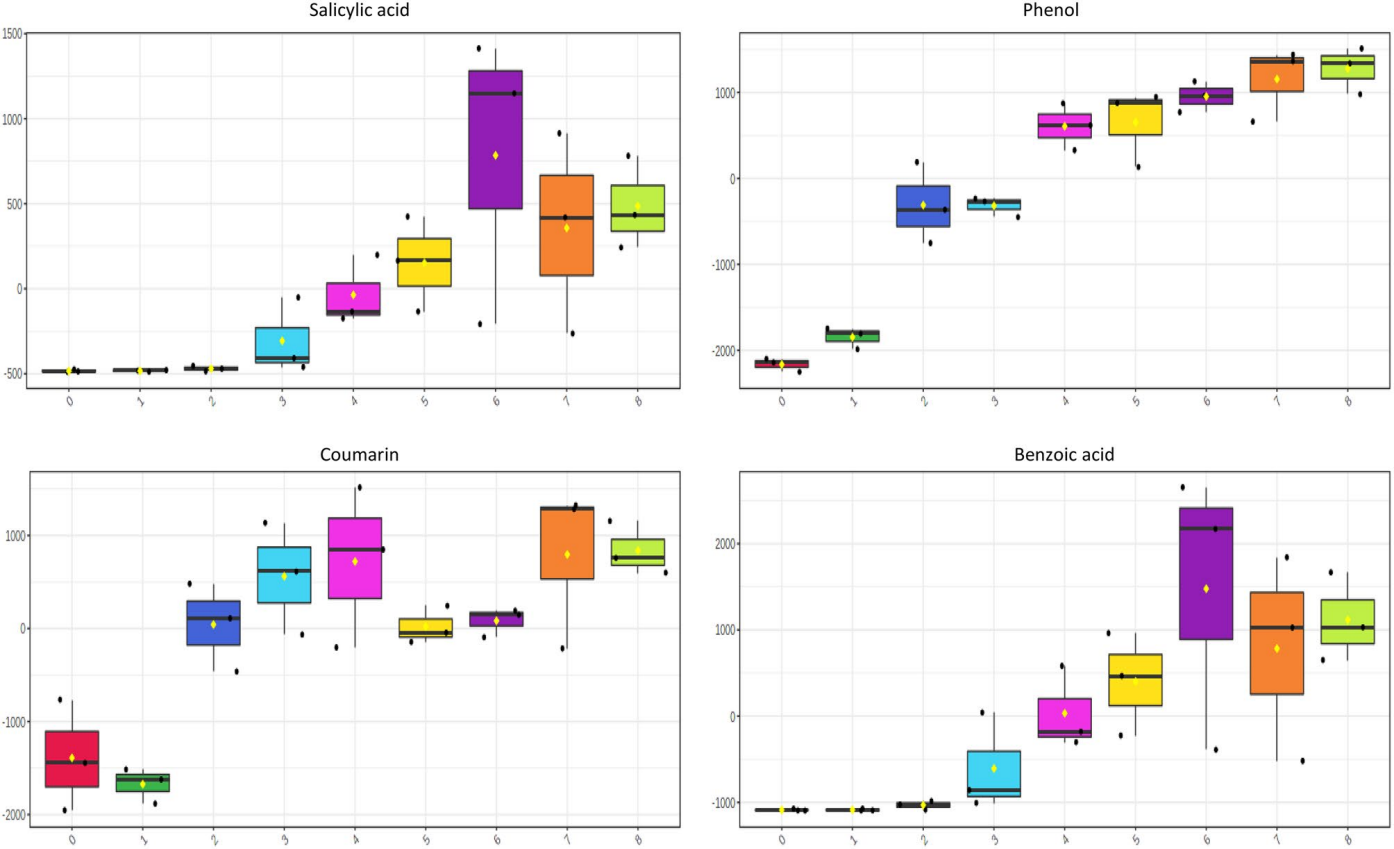
Box plots of metabolites linked with undesirable cocoa sensorial notes with significant differences throughout fermentation days (one-way ANOVA correcting for false discovery rate, FDR). Salicylic acid (phenolic, faint), phenol (phenolic), benzoic acid (urine, faint), coumarin (green, bitter).

To better understand the development of some sensorial notes associated with fine-flavor cocoa (i.e., fruity, floral, and chocolate notes), we performed a search in the database FlavorDB^53^ using the list of altered and annotated compounds. We found a rise between the exothermic and isothermic phases of cedrol, irone, acetophenone, coumaric acid, maltol, vanillin, vanillin isobutyrate, and methylcoumarin (Fig. 7) associated with fine-flavor cocoa sensorial notes^5,53^. Nevertheless, we also observed a significant increase in phenol, coumarin, benzoic acid, and salicylic acid. These molecules produce undesirable flavors previously associated with green, astringent, and bitter attributes (Fig. 8)^5,53^.

**Figure 9:**
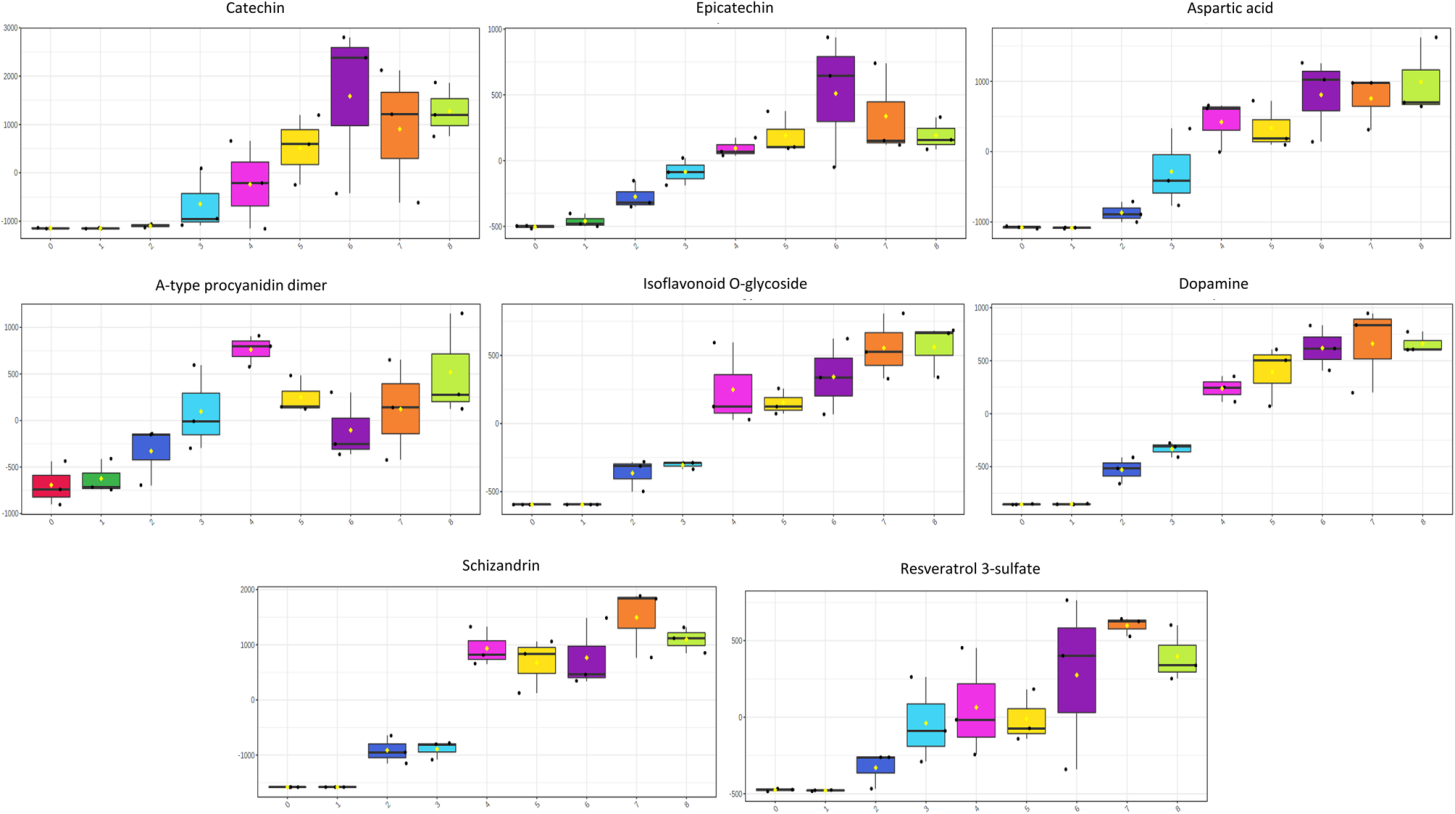
Box plots of bioactive metabolites with significant differences throughout fermentation days (one-way ANOVA correcting for false discovery rate, FDR).

Interestingly, we also observed an increase in some specific flavonoids with documented bioactive properties from the exothermic to isothermic phase. For example, catechin, epicatechin, Resveratrol 3-sulfate, Isoflavonoid *O*-glycoside, and a group of procyanidins dimers and trimers experienced a considerable increase over fermentation (Fig. 9). These compounds are widely known due to their antioxidant properties^8,57^. Similarly, other metabolites as dopamine, nicotinamide riboside, and aspartic acid were observed to increase throughout cocoa fermentation. An attractive potential emerges from these molecules because they can act as neurotransmitters^59^, cardiovascular regulator^60^, and hormone regulator^61^, respectively. These bioactive molecules could be produced from complex polyphenol degradation processes that can involve the action of weak acids and the temperature changes into the beans, oxidation reactions, and hydrolysis by polyphenol oxidases^62,63^ or in the secondary metabolism of some microorganisms^64^, but the biochemical mechanisms behind their production remain unclear.

Several studies associate non-conventional microorganisms’ groups with the production of flavor molecules^64–67^. For instance, unconventional strains of *Saccharomyces*, *Candida*, *Pseudomonas,* and *Bacillus* species, widely reported in cocoa fermentations worldwide, can also produce vanillin and derivates from feluric acid^65,66^. Different fungi species produce coumarin and derivates naturally^64,67^. However, to fully connect the metabolite production with the microbiome of cocoa fermentation, an integration of metabolomics and metagenomic data is required, and it will be the focus of future research.

## Conclusions

Our work reveals a clear connection between cocoa mass temperature and the metabolic activity during the fermentation of fine-flavor cocoa. Using temperature dynamic as a relevant parameter during cocoa fermentation, we proposed a new cocoa fermentation metabolic paradigm that offers a complete insight into how temperature regulates biochemical reactions during cocoa fermentation, considering global metabolic activity. This shift is a crucial step to develop strategies based on temperature control to drive cocoa fermentations toward better quality chocolate. Nevertheless, further research is required to further dissect the link between microorganisms and metabolites. This is a crucial step in order to understand the impact in the sensorial profile of a significant number of cocoa compounds.

We also elucidated metabolic modifications throughout fermentation associated with proteolysis and secondary metabolism. Our results reveal a potential for bioprospection beyond chocolate production of some peptides and polyphenols with attractive bioactive properties that arise during cocoa fermentation—considering that a significant proportion of these molecules are lost in post-fermentation processes. However, the bioactive properties of most of these identified metabolites also still need to be entirely dissected.

## Supplementary Information

Supplementary Information.

## Data Availability

All data generated or analyzed during this study are included in this published article (and its Supplementary Information files).
