# Supplementary material for: Dissecting fine-flavor cocoa bean fermentation through metabolomics analysis to break down the current metabolic paradigm
Source: Sci Rep. 2021 Nov 9;11:21904. doi: 10.1038/s41598-021-01427-8 (PMC8578666; doi:10.1038/s41598-021-01427-8)
Supplement: Supplementary file 1 — Supplementary Information. [file 41598_2021_1427_MOESM1_ESM.docx]

**DISSECTING FINE-FLAVOR COCOA BEAN FERMENTATION THROUGH METABOLOMICS ANALYSIS TO BREAK DOWN THE CURRENT METABOLIC PARADIGM**

Fabio Herrera-Rocha, Mónica P. Cala, Jenny Lorena Aguirre Mejía, Claudia M. Rodríguez-López, María José Chica, Héctor Hugo Olarte, Miguel Fernández-Niño, Andrés Fernando González Barrios.

**SUPPLEMENTARY INFORMATION**

**
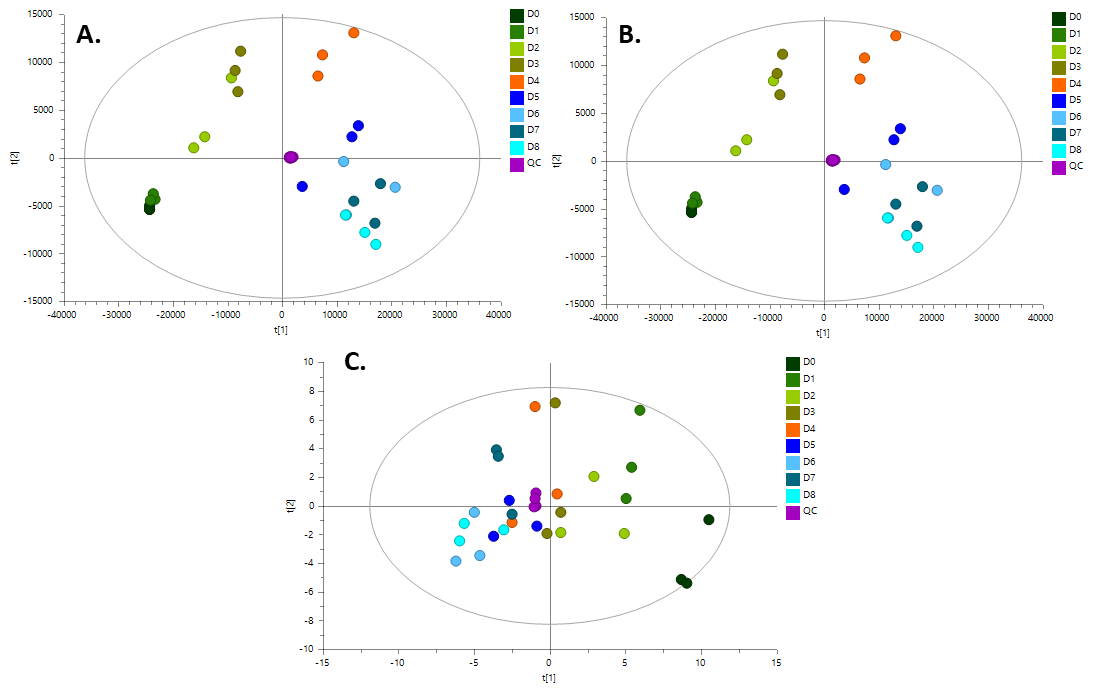
**

**Supplementary Figure S1.** PCA score plots for all fermentation samples (from Day 0 to Day 8) and quality controls (QC) samples analysed with different analytical platforms. **A.** metabolic fingerprinting (MF) by LC-MS(+): R2(cum): 0.875, Q2 (cum): 0.826; **B.** MF by LC-MS(-): R2(cum): 0.876, Q2 (cum): 0.77; **C.** MF by GC-MS: R2(cum): 0.724, Q2 (cum): 0.229.
